# A Prospective Study on Lactate Dehydrogenase Levels in Acute Intestinal Obstruction as a Marker of Bowel Gangrene

**DOI:** 10.7759/cureus.67980

**Published:** 2024-08-27

**Authors:** Anandapadmanabhan Prathapan, Arun Walwekar, Ishwar R Hosamani, Rekha Walwekar

**Affiliations:** 1 General Surgery, Believers Church Medical College Hospital, Thiruvalla, IND; 2 General Surgery, Karnataka Institute of Medical Sciences, Hubli, IND; 3 Pharmacology, Jagadguru Gangadhar Mahaswamigalu Moorusavirmath Medical College, Hubli, IND

**Keywords:** biomarkers, prospective study, acute intestinal obstruction, lactate dehydrogenase levels, bowel gangrene

## Abstract

Background

Intestinal obstruction continues to be one of the most common causes of emergencies presenting to the surgical department. A thorough clinical examination along with relevant investigations continue to be best modality for timely management and prompt decision-making in such cases.

Objectives

The aim of this study was to identify patients of intestinal obstruction with elevated levels of serum lactate dehydrogenase (LDH) and correlate elevated LDH levels with bowel viability.

Materials and methods

The study comprised 46 cases of intestinal obstruction and their serum LDH values measured prior to surgical intervention/first day of admission. The intra-operative bowel viability was noted and compared with LDH values obtained, and their correlation was studied along with other variables of the patient.

Results

In our study, it was found that a serum LDH level of >800 IU/L signified an abnormal bowel with increased risk of bowel gangrene, as indicated by a sensitivity of 75% and a specificity of 94%. Gangrenous bowel was found to be more associated with causes such as superior mesenteric artery/vein thrombosis, volvulus, and habits such as alcoholism and tobacco consumption. Elevated LDH levels, however, do not correlate with the length of the affected gangrenous bowel, with the duration of presentation of symptoms, or as a predictor of mortality.

Conclusion

Serum LDH can be used as a good predictor of gangrenous bowel in cases of intestinal obstruction.

## Introduction

Intestinal obstruction is one of the most common emergency surgical cases to be presented to a surgeon and poses a challenge to both diagnosis and treatment planning of the patient [1]. Each case presented and rate of progression depend on a multitude of factors including duration of symptoms, cause of obstruction, degree of obstruction, performance status of the patient, geographical variations, and others, and, hence, prompt decision-making is the key to management for each patient [2]. The status of bowel in obstruction plays an important factor in planning operative or non-operative management in each case [3].

Looking at these parameters and the non-availability of a definitive lab investigation for the status of bowel in an obstruction case, clinical examination and radiologic investigations go hand in hand, in which radiologic investigations usually are under time constraints [4]. Hence, a reliable laboratory parameter is needed to assist in the decision-making process [5]. This study aims to find the correlation between serum lactate dehydrogenase (LDH) levels and degree of bowel gangrene in a clinical case of intestinal obstruction, including conversion from non-operative to operative management [6].

LDH enzymes are widely distributed in cells of various living systems and is involved in carbohydrate metabolism, catalyzing inter-conversion of lactate and pyruvate [7]. LDH activity is predominantly seen in the cytoplasm of the cell, and when the intestinal mucosa undergoes tissue hypoxia/ischemia, it is released into the serum and its serum level increases [8].

## Materials and methods

Ethics committee approval had been taken from Karnataka Institute of Medical Sciences Hubballi Ethics Committee (Reg No.- ECR/486/Inst/KA/2013/RR-16), held on 19^th^ November 2019. This is a descriptive study of 46 patients with intestinal obstruction who were admitted between December 2019 and December 2021 to the general surgery department at the KIMS, Hubli.

Sample size

The minimum sample size required for this study was 43 patients with intestinal obstruction [9].

 Sample size calculation ‘n’ = [Z^2^ (p*q)] /d^2^

Where:

n= Sample Size

Z = Z value (1.96 for 95% confidence level)

p = Anticipated percentage of intestinal obstruction cases with bowel gangrene

p = 0.5 (50%)

q = 1 - p

d = Error (15%)

Calculation:

1.96^2^ [0.5 * 0.5] / (0.15)^2^

= 42.67, rounded up to 43.

Inclusion criteria

Patients presenting to KIMS Hubli with features of acute/sub-acute intestinal obstruction and patients with irreducible inguinal or femoral hernias were included in the study.

Exclusion criteria

Patients younger than 18 years and those older than 80 years, pregnant patients, and known cases of liver disease and HIV infection were excluded from the study.

Every patient who showed signs of intestinal obstruction was admitted as an emergency. Full medical history, with particular attention to assessing the classic signs of intestinal obstruction, such as distention, vomiting, and constipation, as well as a history of previous surgeries and tuberculosis were taken. A comprehensive examination to check for dehydration, hemodynamic stability, abdominal distention, hernia orifices, palpable lumps, surgical scars, and bowel sounds was performed afterward [3].

 Following a clinical diagnosis of intestinal blockage, regular examinations as well as other essential investigations were carried out. To determine the degree of obstruction, an X-ray of the abdomen was also performed to look for the air-fluid levels. In most cases, an abdominal ultrasonography was performed. When conservative therapies improved the patient's overall state, it was determined that there was no need for urgent surgical therapy, and in those situations, contrast studies and CT scans were performed [5]. Because manual reduction of an obstructed hernia can lead to problems such as widespread peritonitis from reduction of a gangrenous section into the abdominal cavity, perforation of viscera, and en mass reduction, it is best avoided. A proforma was used to record all of this data. On the other hand, rapid resuscitation was initiated if the patient's hemodynamic stability was compromised. A central venous line was placed, and hypovolemia was treated with a regular saline infusion. Additionally, blood was organized according to hemoglobin levels. A Foley catheter was inserted to monitor urine output, and a nasogastric tube was introduced to decompress the gut [4].

A duration of 24 to 48 hours was observed for nasogastric output, abdominal girth monitoring, bowel sounds, and hemodynamic stability. Nonetheless, an urgent procedure was performed in case obstructive features persisted, in case of a strangulated or obstructed hernia, or in the event of peritonitis symptoms. Third-generation cephalosporins combined with metronidazole were administered at the time of inducing anesthesia [2]. A transverse inguinoscrotal incision was used to treat an obstructed or strangulated inguinal hernia. During handling, every attempt was taken to avoid the spontaneous decrease of the intestine to the peritoneal cavity. The sac's fluid was removed for sensitivity testing and culture, and its potential to leak into the wound was examined. To address other sources of intestinal obstruction, a midline laparotomy incision was made. Site of obstruction was identified by observing the condition of the caecum. If the caecum was distended, cause of obstruction was in the large gut, and if it was collapsed, it was in the small intestine. Underlying pathology was identified and treated accordingly [1]. The data regarding post-operative management were not collected.

Investigations required included serum LDH (LDH was estimated in all the cases preoperatively on the day of admission) [6]. The estimated value of serum LDH was compared with the viability of the bowel intra-operatively, complete hemogram, renal function test, serum electrolytes, blood grouping and cross-matching, random blood sugar screening, HIV, HbsAg, X-ray of the abdomen, ultrasound of the abdomen, CT scan of the abdomen (if required).

## Results

The study included 46 cases of acute intestinal obstruction between December 2019 and December 2021. Following admission, every case was monitored during surgical management, and all pertinent information was documented. Post-operative follow-up was not part of the study. This was summarized into a master chart. The comparable tabulations permit certain statistical inferences to be made and are presented below.

Etiology of obstruction

In this study, the most frequent reason for sudden intestinal obstruction was a hernia. It represented 32.6% of the total cases. 10 (21.7%) instances had obstruction from adhesions or bands, five (10.9%) cases had cancer, four (8.7%) cases had sigmoid volvulus, one (2.2%) case had ileocecal tuberculosis, one (2.2%) case had superior mesenteric artery/vein thrombosis, three (6.8%) cases had strictures, one (2.2%) case had fecal impaction, and four (8.7%) cases had an unknown cause. All of these patients were treated conservatively (Table 1).

**Table 1:** Etiology of obstruction of the study population (n=46) SMA, superior mesenteric artery; SMV, superior mesenteric vein

Etiology	No. of cases (n)	Percentage (%)
Obstructed hernia	15	32.6%
Adhesions/bands	10	21.7%
Malignancy	5	10.9%
Sigmoid volvulus	4	8.7%
SMA thrombosis	2	4.3%
Strangulated hernia	2	4.3%
Unknown etiology	4	8.7%
Fecal impaction	1	2.2%
Intestinal tuberculosis	1	2.2%
SMV thrombosis	1	2.2%
Stricture	1	2.2%

Age, gender distribution of the study population, and incidence

Intestinal obstruction can occur at any age, as the results of this show. The age range that was most frequently impacted was 51-65 years, and males were more prevalent in each age group. Average age of presentation of acute intestinal obstruction was 49.6 years (p-value for sex distribution was 0.50055, which is statistically not significant). The most common age of presentation of cases was 51-65 years, with a p-value of 0.696, which is statistically not significant. Intestinal obstruction cases were associated more with the male sex than the female sex and showed a p-value of 0.606, which was statistically not significant (Table 2).

**Table 2:** Age and gender distribution of the study population (n=46)

Age range (years)	Male (n)	Percentage (%)	Female (n)	Percentage (%)	Total (n)	Percentage (%)
20-35	8	26.7%	4	25.0%	12	26.1%
36-50	6	20.0%	6	37.5%	12.2	26.5%
51-65	11	36.7%	3	18.8%	14	30.4%
65-80	5	16.7%	3	18.8%	8	17.4%
Total	30	100.0%	16	100.0%	46	100.0%

Clinical symptomatology

In nearly all cases (43 cases, 93.4%), there was abdominal pain. In 25 (54.3%) cases, there was vomiting. In 13 (28.2%) cases, there was abdominal distension. In certain circumstances, the early presentation may be the reason for the lack of distension. Also, 16 (34.7%) patients complained of constipation (Table 3).

**Table 3:** Clinical symptomatology

Symptoms	No. of cases (n)	Percentage (%)
Abdominal pain	43	93.4%
Vomiting	25	54.3%
Abdominal distension	13	28.2%
Obstipation/constipation	16	34.7%

Duration of presenting complaints

In 32 (69.6%) patients, duration of presentation was one to five days, in eight (17.4%) patients, it was six to nine days, and in six (13%) patients, it was more than 10 days, with a mean (SD) of 4.65 (4.37) days and a median of 3 (2-7) days (Table 4).

**Table 4:** Duration of presenting complaints (n=46)

Duration of presentation	No. of cases (n)	Percentage (%)
1-5 days	32	69.6%
6-9 days	8	17.4%
>10 days	6	13.0%

Comorbidities in the study population

Of the 45 patients, 36 (78.3%) patients had no comorbidities, four (8.7%) patients had hypertension, three (6.5%) patients had tuberculosis, one (2.2%) patient had diabetes mellitus, one (2.2%) patient had seizure disorder, and one (2.2%) patient had ischemic heart disease (IHD) as a comorbidity (Table 5).

**Table 5:** Comorbidities in the study population (n=46) IHD, ischemic heart disease

Comorbidities	No. of cases (n)	Percentage (%)
No comorbidities	36	78.3%
Hypertension	4	8.7%
Tuberculosis	3	6.5%
Diabetes mellitus	1	2.2%
Seizure disorder	1	2.2%
IHD	1	2.2%

Habits of the study population

Out of 45 patients, 33 (71.7%) patients had no history of chronic alcohol consumption or smoke or chewing tobacco, five (10.9%) patients were chronic alcoholic, three (6.5%) patients were chronic tobacco chewer, two (4.3%) patients were chronic alcoholic and tobacco chewer, two (4.3%) patients were chronic smoker, and one (2.2%) patient was chronic alcoholic and smoker (Table 6).

**Table 6:** Habits of the study population (n=46)

Habits	No. of cases (n)	Percentage (%)
No habits	33	71.7%
Chronic alcoholic	5	10.9%
Chronic tobacco chewer	3	6.5%
Chronic alcoholic and tobacco chewer	2	4.3%
Chronic smoker	2	4.3%
Chronic alcoholic and smoker	1	2.2%

Smoking and alcohol habits versus bowel gangrene

Seven (15.5%) patients with a habit of smoking and drinking alcohol developed bowel gangrene, with a p-value of 0.0407, which is statistically significant, indicating that patients with smoking and alcohol habits had a higher risk of developing bowel gangrene (Table 7).

**Table 7:** Smoking and alcohol habits versus bowel gangrene

Habits	Present (n)	Percentage (%)	Absent (n)	Percentage (%)
Present	7	15.5	6	13.3
Nil	7	15.5	26	57.7
Total	14	31.1	32	71.1

Abdominal signs

In total, 14 (30.4%) patients had tachycardia, 40 (86.9%) patients had abdomen tenderness, 11 (23.9%) patients had guarding/rigidity, 16 (34.7%) patients had no bowel sound, and 15 (32.6%) patients had a previous surgical scar (Table 8).

**Table 8:** Abdominal signs

Signs	No. of cases (n)	Percentage (%)
Tachycardia	14	30.4%
Tenderness	40	86.9%
Guarding/rigidity	11	23.9%
Bowel sounds (absent)	16	34.7%
Previous surgical scar	15	32.6%

Erect X-ray of the abdomen

In erect X-ray of the abdomen, multiple fluid levels were present in 15 (32.6%) patients, dilated bowel loops were present in 12 (26%) patients, and coffee bean appearance was present in 15 (32.6%) patients (Table 9).

**Table 9:** Erect X-ray of the abdomen

Findings	No. of cases (n)	Percentage (%)
Multiple fluid levels	15	32.6%
Dilated bowel loops	12	26%
Inconclusive	4	8.6%
Coffee bean appearance	15	32.6%

Bowel status when compared to etiology of the study population

This comparison showed that bowel gangrene was seen more in patients with volvulus and SMA/SMV thrombosis, with a p-value of 0.000573, which is statistically significant (chi-square test=80.9) (Table 10).

**Table 10:** Bowel status when compared to etiology of the study population (n=46) SMA, superior mesenteric artery; SMV, superior mesenteric vein

Etiology	Dilated	Gangrenous	Ischemic/congested	Managed conservatively	Normal	Total, n (%)
Obstructed hernia	11	3	-	-	1	15 (32.6)
Adhesions/bands	8	2	-	-	-	10 (21.7)
Malignancy	5	-	-	-	-	5 (10.8)
Sigmoid volvulus	-	3	1	-	-	4 (8.6)
SMA thrombosis	-	2	-	-	-	2 (4.3)
Strangulated hernia	1	1	-	-	-	2 (4.3)
Unknown	2	-	-	1	1	4 (8.6)
Fecal impaction	-	-	-	1	-	1 (2.1)
Intestinal tuberculosis	-	-	1	-	-	1 (2.1)
SMV thrombosis	-	1	-	-	-	1 (2.1)
Stricture	1	-	-	-	-	1 (2.1)
Total	28	12	2	2	2	46 (100)

Comorbidities when compared to bowel gangrene

This comparison showed that comorbidities are not an influencing factor in the presence of bowel gangrene, with a p-value of 0.15 (statistically not significant) and a chi-square of 8.109 (Table 11).

**Table 11:** Comorbidities when compared to bowel gangrene (n=46) IHD, ischemic heart disease

Comorbidities	Absent	Present	Total, n (%)
No comorbidities	26	10	36 (78.2)
Hypertension	1	4	5 (10.8)
Tuberculosis	2	-	2 (4.3)
Diabetes mellitus	1	-	1 (2.1)
Seizure disorder	1	-	1 (2.1)
IHD	1	-	1 (2.1)
Total	32	14	46 (100)

Duration of symptoms versus the presence of gangrene

This comparison showed that duration of symptoms was not a risk factor in patients with bowel gangrene, with a p-value of 0.383 (statistically not significant) (Table 12).

**Table 12:** Duration of symptoms versus the presence of gangrene (n=46)

Duration of symptoms	Gangrene present, n (%)	Gangrene absent, n (%)
1-5 days	8 (17.3)	24 (52.1)
6-9 days	4(8.6)	4 (8.6)
>10 days	2 (4.3)	4 (8.6)
Total	14 (30.4)	32 (69.5)

Procedures

Reduction and hernia repair was performed in 13 (28.2%) cases, resection and anastomosis in 19 (41.3%) cases, adhesiolysis/band release in seven (15.2%) cases, colostomy/ileostomy in three (6.5%) cases, Hartmann’s procedure in three (6.5%) cases, and conservative management was used in three (6.5%) cases (Table 13).

**Table 13:** Procedures

Procedure	No. of cases (n)	Percentage (%)
Reduction and hernia repair	13	28.2%
Resection and anastomosis	19	41.3%
Adhesiolysis/band release	7	15.2%
Colostomy/ileostomy	3	6.5%
Hartmann’s procedure	3	6.5%
Managed conservatively	3	6.5%

LDH levels in comparison to etiology bowel status of the study population

Overall, 28 patients had dilated bowel: three (6.5%) patients had 50-200 IU/L of LDH levels, 20 (43.5%) patients had 200-400 IU/L, four (8.7%) patients had 400-800 IU/L, and one (2.17%) patient had >1,200 IU/L LDH. In addition, 12 patients had gangrenous bowel: six (13.04%) patients had 800-1,200 IU/L of LDH levels and six (13.04%) patients had >1,200 IU/L. Two patients had ischemic bowel: one (2.17%) patient had 400-800 IU/L of LDH levels and one (2.17%) patient had 800-1,200 IU/L. two patients had normal bowel: one (2.17%) patient had 200-400 IU/L of LDH levels and one (2.17%) patient had 400-800 IU/L. A comparison of LDH levels with bowel gangrene showed a p-value of 0.000057, which was statistically significant, which implied that raised LDH levels indicate increased risk of bowel gangrene (Table 14).

**Table 14:** LDH levels in comparison to etiology of bowel status of the study population (n=46) LDH, lactate dehydrogenase

LDH levels (IU/L)	Dilated	Gangrenous	Ischemic	Managed conservatively	Normal
50-200	3 (6.5%)	-	-	-	-
200-400	20 (43.5%)	-	-	1 (2.17%)	1 (2.17%)
400-800	4 (8.7%)	-	1 (2.17%)	1 (2.17%)	1 (2.17%)
800-1,200	-	6 (13.04%)	1 (2.17%)	-	-
>1,200	1 (2.17%)	6 (13.04%)	-	-	-

LDH levels in comparison to length of the gangrenous bowel

This comparison evaluated LDH levels with the length of the involved bowel and showed a p-value of 0.371, which was statistically insignificant, which meant that LDH levels do not correlate with length of the involved bowel (Table 15).

**Table 15:** LDH levels in comparison to length of the gangrenous bowel (n=14) LDH, lactate dehydrogenase

LDH levels	Length of gangrenous bowel (in cm), n (%)		
	<20	21-40	>40
50-200	-	-	-
200-400	-	-	-
400-800	-	1 (2.1%)	-
800-1,200	5 (10.8%)	1 (2.1%)	1 (2.1%)
>1,200	-	4 (8.6%)	2 (4.3%)

LDH levels in comparison to outcome of patients with gangrene

This comparison evaluated the LDH level with mortality of the patient, which showed a p-value of 0.976, which is statistically insignificant. Thus, it is not a good predictor of mortality in patients with bowel gangrene (Table 16).

**Table 16:** LDH levels in comparison to outcome of the patients with gangrene (n=14) LDH, lactate dehydrogenase

LDH level	Outcome	
	Mortality	No mortality
50-200	0	0
200-400	0	0
400-800	0	1
800-1,200	2	5
>1,200	2	4
Total	4 (28.57%)	10 (71.42%)

Binary logistic analysis of LDH versus gangrene

A logistic regression was performed to ascertain the effects of LDH on the likelihood that participants have gangrenous bowel. The logistic regression model was statistically significant, with χ2(4) = 47.45, p < 0.0000579, degree of freedom 10 (Table 17).

**Table 17:** Binary logistic analysis of LDH versus gangrene LDH, lactate dehydrogenase

Hosmer and Lemeshow test		
Chi-square	df	Sig.
47.45	10	<0.0000579

LDH levels in comparison to erect X-ray of the abdomen of the study population

On erect X-ray of the abdomen, 15 patients had multiple fluid levels: three (6.52%) patients had 50-200 IU/L of LDH levels, six (13.04%) patients had 200-400 IU/L, four (8.69%) patients had 400-800 IU/L, one (2.17%) patient had 800-1,200 IU/L, and one (2.17%) patient had >1,200 IU/L LDH. Fifteen patients had coffee bean appearance (CBA): six (13.04%) patients had 200-400 IU/L of LDH levels, one (2.17%) patient had 400-800 IU/L, five (10.86%) patients had 800-1,200 IU/L, and three (6.52%) patients had >1,200 IU/L of LDH levels. Twelve patients had dilated bowel loops (DBL): 10 (21.73%) patients had 200-400 IU/L of LDH levels, one (2.17%) patient had 400-800 IU/L, and one (2.17%) patient had 800-1,200 IU/L. Four patients had inconclusive X-ray findings: three (6.52%) patients had 200-400 IU/L of LDH levels and one (2.17%) patient had 400-800 IU/L. A comparison of LDH levels with X-ray findings showed a p-value of 0.057, which was statistically not significant (Table 18).

**Table 18:** LDH levels in comparison to erect X-ray of the abdomen of the study population (n=46) LDH, lactate dehydrogenase

LDH levels (IU/L)	Erect abdominal X-ray findings			
	Multiple fluid levels (15)	Coffee bean appearance (15)	Dilated bowel loops (12)	Inconclusive (4)
50-200	3 (6.52%)	-	-	-
200-400	6 (13.04%)	6 (13.04%)	10 (21.73%)	3 (6.52%)
400-800	4 (8.69%)	1 (2.17%)	1 (2.17%)	1 (2.17%)
800-1,200	1 (2.17%)	5 (10.86%)	1 (2.17%)	-
>1,200	1 (2.17%)	3 (6.52%)	-	-

In intestinal obstruction, it is frequently necessary to use radiological interventions, such as CT, ultrasound, or X-rays. The most effective method for diagnosis and determining the underlying severity of intestinal obstruction is contrast-enhanced CT (CECT). However, CECT is frequently not an option since patients arrive at the hospital late and often have sepsis, shock, and multiorgan failure, particularly in the kidneys, for which contrast-enhanced radiological studies are not advised.

## Discussion

A total of 46 cases of intestinal obstruction were included in the two-year study based on clinical evaluation and radiographic evidence of acute intestinal obstruction at the KIMS Hubli.

In this study, cases who presented with features of intestinal obstruction and whose LDH levels were determined prior to operative procedures were selected, totaling 46 cases. Throughout their hospital stay, every patient was monitored, and pertinent information was recorded in the master chart for comparison and statistical analysis. Patients with smoking and alcohol habits had a higher risk of developing bowel gangrene.

In this study, the mean age was 49.6 years. The majority of the patients were males (n=30, 65.2%) and the rest were females (n=16, 34.7%), but no statistical significant difference was observed, which was comparable to other studies conducted by Rajaashok et al. [10] and Nitesh Soni et al. [11]. These studies showed no statistical significance in bowel gangrene in comparison to age or sex of the patient.

In this study, 43 (93.4%) patients reported having abdominal pain, while the remaining three (6.5%) patients did not report any abdominal pain. Of the patients, 32.6% (n = 15) had irreducible hernia, whereas the remaining 31 (67.3%) patients did not. Also, 54.3% of patients (n = 25) experienced vomiting, whereas 45.6% of patients (n = 21) did not experience vomiting. Of the 16 cases, 34.7% (n=16) had constipation or diarrhea, whereas the remaining cases did not. In 28.2% (n=13) of the cases, abdominal distension was evident, but in 71.7% (n=33) of the cases, it was not. Stiffness and guarding were absent in 35 (76%) of the cases, but guarding was present in 23.9% (n=11) of the cases, while the rest (n=35, 76%) did not have guarding or rigidity. Thirty (65.2%) patients had bowel sounds when they first presented, compared to 16 (34.7%) patients who did not. The majority of them (n=32, 9.6%) exhibited symptoms for fewer than five days. Similar studies by Soni et al. [11], Mucha [12], and Rajaashok et al. [10] reported results that were similar to these. Approximately 17.4% (n=8) of the participants experienced symptoms for six to nine days, while the remaining 13% (n=6) had symptoms for more than 10 days.

In this study, intestinal gangrene was evident in 26% (n=12) of the cases; the remaining instances were either in an ischemic state (n=2, 4.3%) or in a normal state (n=32, 69.5%). The most common cause of intestinal obstruction was hernia (32%) followed by post-operative adhesions or band formation (19%), which was in contrast to other similar studies conducted by Rajaashok et Al. [10], which showed the most common cause as adhesions as the most common cause (46%), followed by obstructed hernias (23%).

In this study, the mean serum LDH was high (mean=1022) in patients with gangrene. An LDH level of 400-800 IU/L was highly suspicious of gangrene or pre-gangrenous changes (6.6%), an LDH level of 800-1,200 IU/L was highly suggestive of bowel gangrene (85%), and levels >12,00 IU/L showed apparent gangrenous changes (85%), which was supported with a statistical significance in p-value (<0.05). Studies conducted by Lange and Jackel et al. [13] and Block et al. [14] revealed an inference of 100% sensitivity and 42% specificity for increased LDH in patients presenting with acute abdomen who were later found to have ischemia and gangrene.

In this study, the length of gangrenous bowel involved, however, was not correlating with levels of LDH, with affected segment of more than 200 cm of bowel showing LDH levels in the range of 800-1,500 and gangrenous bowel segments less than 50 cm showed LDH varying between 800 and 1,500 similarly, with a p-value of >0.05, which was comparable to other studies conducted by Rajaashok et al. [10], Mucha et Al. [12], Manjunath et al. [15], Lange and Jackel [13], and Balakrishna et al. [16]. The presence of gangrene was associated more in patients who presented with a shorter duration of symptoms (one to five days, 57%), while patients presenting with 10 days’ duration of symptoms showed a lower rate of bowel gangrene (28%), but this was statistically insignificant (Table 18).

**Table 19:** Comparison of sensitivity and specificity of LDH values with other studies

Study	Sensitivity	Specificity	Significant LDH value
Present study	75%	94%	>800 IU/L
Soni et al. [11]	88%	68%	>800 IU/L
Manjunath et al. [15]	88%	76%	>700 IU/L
Rajaashok et al. [10]	80%	82%	>1,000 IU/L
S V Balakrishna et al. [16]	80%	80%	>1,000 IU/L
Lange et al. [13]	100%	42%	>1,000 IU/L
Mucha [12]	80%	90%	>1,000 IU/L

It is frequently necessary to use radiological interventions, such as CT, ultrasound, or X-ray of the abdomen, when there is intestinal obstruction. The most effective method of examination for making a diagnosis and determining the underlying severity of the illness is CECT. However, CECT is frequently not an option as patients arrive at the hospital late and often have sepsis, shock, and multiorgan failure, particularly in the kidneys, for which contrast-enhanced radiological studies are not advised. Therefore, the severity cannot be determined until the patient has undergone surgery, and the intra-operative results have been reviewed. Therefore, in order to fully prepare for the outcomes and hazards of intra- and post-operative care, additional investigative tools are needed for the preoperative assessment of the severity of the underlying pathology. Regarding this, using serum LDH in all patients presenting with acute abdomen, who can be screened prior to surgery, not only provides a crucial hint about the underlying pathology and severity of intestinal necrosis but also allows the surgeons to plan the surgery appropriately and inform patients and their families about what to expect during and after the procedure [15]. Testing for LDH is a less invasive, cost-effective, and easily available diagnostic tool to diagnose bowel ischemia/gangrene. Hence, it is more useful in centers where diagnostic facilities are limited.

This study was conducted in a single institution, and the small sample size was a limitation. Further multicentric studies with large sample size are recommended.

## Conclusions

This study on intestinal obstruction was aimed at identifying the association between levels of serum LDH and the presence of bowel gangrene. It was found that a serum LDH level of >800 IU/L signified an abnormal bowel with an increased risk of bowel gangrene, as indicated by a sensitivity of 75% and a specificity of 94%, highlighting the increased probability of abnormal bowel (congested/ischemic/gangrenous changes). Raised serum LDH levels aid in identifying the occurrence and progression of intestinal obstruction to gangrene. From this study, it is evident that ischemic changes in any part of the bowel can cause elevation in the serum levels of LDH, and a higher value of >800 IU/L strongly indicates an underlying gangrenous change.
